# Simazine Enhances Dark Fermentative H_2_ Production by Unicellular Halotolerant Cyanobacterium *Aphanothece halophytica*

**DOI:** 10.3389/fbioe.2022.904101

**Published:** 2022-07-15

**Authors:** Sunisa Pansook, Aran Incharoensakdi, Saranya Phunpruch

**Affiliations:** ^1^ Department of Biology, School of Science, King Mongkut's Institute of Technology Ladkrabang, Bangkok, Thailand; ^2^ Laboratory of Cyanobacterial Biotechnology, Department of Biochemistry, Faculty of Science, Chulalongkorn University, Bangkok, Thailand; ^3^ Bioenergy Research Unit, School of Science, King Mongkut's Institute of Technology Ladkrabang, Bangkok, Thailand

**Keywords:** H_2_ production, cyanobacteria, *Aphanothece halophytica*, inhibitor, simazine

## Abstract

The halotolerant cyanobacterium *Aphanothece halophytica* is a potential H_2_ producer that induces H_2_ evolution under nitrogen deprivation. H_2_ is mainly produced *via* the catabolism of stored glycogen under dark anaerobic condition. H_2_ evolution is catalyzed by O_2_-sensitive bidirectional hydrogenase. The aim of this study was to improve H_2_ production by *A. halophytica* using various kinds of inhibitors. Among all types of inhibitors, simazine efficiently promoted the highest H_2_ production under dark conditions. High simazine concentration and long-term incubation resulted in a decrease in cell and chlorophyll concentrations. The optimal simazine concentration for H_2_ production by *A. halophytica* was 25 µM. Simazine inhibited photosynthetic O_2_ evolution but promoted dark respiration, resulting in a decrease in O_2_ level. Hence, the bidirectional hydrogenase activity and H_2_ production was increased. *A. halophytica* showed the highest H_2_ production rate at 58.88 ± 0.22 µmol H_2_ g^−1^ dry weight h^−1^ and H_2_ accumulation at 356.21 ± 6.04 μmol H_2_ g^−1^ dry weight after treatment with 25 µM simazine under dark anaerobic condition for 2 and 24 h, respectively. This study demonstrates the potential of simazine for the enhancement of dark fermentative H_2_ production by *A. halophytica*.

## 1 Introduction

Taking into consideration the concern of limited fossil fuel and the environmental impact of energy consumption, great attention has been paid to the renewable energy sources for a replacement of primitive fossil fuels. H_2_ is an interesting alternative renewable energy carrier. The combustion of H_2_ yields a high heating value of 141.6 MJ kg^−1^ (Perry, 1963). Due to the chemical structure of H_2_ as a non-carbon–based molecule, H_2_ burning does not emit greenhouse gases or other pollutants into the environment. H_2_ is mainly produced by chemical processes; however, it can be produced by various kinds of organisms *via* many metabolic pathways depending on the organism types (Levin et al., 2004). Cyanobacteria and green algae can generate H_2_ using electrons obtained from photosynthetic electron transport and/or from stored glycogen degradation during dark fermentation (Srirangan et al., 2011).

The unicellular halotolerant cyanobacterium *Aphanothece halophytica* is a potential H_2_ producer (Taikhao et al., 2013). It can grow in strong external NaCl concentrations up to 3 M NaCl (Takabe et al., 1988) and in abundant natural seawater supplemented with merely 1.76 mM NaNO_3_ (Taikhao et al., 2015). H_2_ production by *A. halophytica* is notably induced under nitrogen deprivation by a catabolism of stored glycogen under anaerobic condition in darkness but is hardly detected under anaerobic condition in the light (Taikhao et al., 2013, 2015). H_2_ evolution by *A. halophytica* is catalyzed by the bidirectional hydrogenase (Phunpruch et al., 2016), which is sensitive to molecular oxygen evolved by the water-splitting reaction at photosystem II during photolysis reaction (McKinlay and Harwood, 2010). Moreover, sustainable long-term H_2_ production is enhanced in *A. halophytica* cells immobilized with agar and alginate (Pansook et al., 2016; 2019a).

H_2_ is chemically evolved through a reduction of protons by electrons. In cyanobacteria, electrons are generated by photosynthetic pathways using water as an electron donor. Electrons can be transferred to many chemical reactions through various metabolic pathways, such as CO_2_ fixation, carbohydrate metabolism, and the respiratory electron transport chain (Eroglu and Melis, 2011; Srirangan et al*.*, 2011). To enhance H_2_ production, using inhibitors that hinder electron transfer to other processes is a choice. Consequently, more electrons are directly transferred to bidirectional hydrogenase to produce H_2_. Several inhibitors with the ability to direct electrons toward H_2_ metabolism of cyanobacteria are photosystem II inhibitor, respiratory inhibitor, uncoupling agent of oxidative phosphorylation inhibitor, CO_2_ fixation inhibitor, and Krebs cycle inhibitor. In *Anabaena* spp. strains CA and 1F, *Anabaena cylindrica*, and *Anabaena* sp. PCC7120, H_2_ production increases after treatment with photosystem II inhibitor 3-(3,4-dichlorophenyl)-1,1-dimethylurea (DCMU) under light (Zhang et al., 1983; Chen et al., 2013; Chen et al., 2014). Moreover, Krebs cycle inhibitor malonate also increases H_2_ production in *Synechocystis* sp. PCC6803 and *Anabaena siamensis* TISTR 8012 (Burrows et al., 2011; Khetkorn et al., 2012).

In this study, screening for inhibitors of H_2_ production by *A. halophytica* were investigated. Simazine was found to be a potential inhibitor for H_2_ production by *A. halophytica* under both light and dark conditions. Then, the effect of simazine concentration on cell concentration, chlorophyll *a* content, and H_2_ and O_2_ production was investigated. Finally, bidirectional hydrogenase activity, PSII, and dark respiration activities by *A. halophytica* treated with various concentrations of simazine were also investigated.

## 2 Materials and Methods

### 2.1 Cyanobacterial Growth Condition

*A. halophytica* was grown in a 250-ml Erlenmeyer flask containing 100 ml of BG11 (pH 7.4) (Rippka et al., 1979) supplemented with Turk Island salt solution (Garlick et al., 1977). *A. halophytica* cells were cultivated with an initial OD_730_ of approximately 0.1 and shaken at 120 rpm, 30°C under a cool white-light intensity of 30 µmol photons m^−2^ s^−1^ (16 h light and 8 h dark days^−1^) for 7 days.

### 2.2 Screening of Inhibitors Affecting H_2_ Production by *A. halophytica*

*A. halophytica* grown as previously described for 7 days was harvested by centrifugation at 8,000 × g at 4°C for 10 min, subsequently washed twice, and finally resuspended in 100 ml of nitrogen-free BG11 (BG11_0_) supplemented with Turk Island salt solution. Cells were shaken on a rotary shaker at 120 rpm at 30°C under a white-light intensity of 30 µmol photons m^−2^ s^−1^ for 24 h. Cells were then harvested by centrifugation, resuspended in 5 ml of BG11_0_ supplemented with Turk Island salt solution, and transferred to a 10-ml glass vial. Various kinds of inhibitors comprising photosystem II inhibitors such as atrazine (2-chloro-4-ethylamino-6-isopropylamino-1,3,5-triazine) (Sigma, Germany), DCMU [3-(3,4-dichlorophenyl)-1,1-dimethylurea] (Sigma, Germany), glyphosate [N-(phosphonomethyl)-glycine] (Sigma, Germany), and simazine (2-chloro-4,6-diethylamino-1,3,5-triazine) (Sigma, Germany); respiration inhibitors such as malonic acid (Sigma, Germany), rotenone (Sigma, Germany), and sodium azide (Sigma, Germany); an inhibitor of uncoupling agent of oxidative phosphorylation 2,4-dinitrophenol (DNP) (Sigma, Germany); a CO_2_ fixation inhibitor glyceraldehyde (Sigma, Germany); and a Krebs cycle inhibitor sodium arsenate (Sigma, Germany) at a final concentration of 5 µM were added into the cell suspension. The vials were sealed with a rubber stopper and further incubated at 30°C under the light for 2 h. Then, the vials were purged with argon gas for 10 min and incubated at 30°C under light or in darkness for 2 h before H_2_ measurement. In this study, simazine as an effective inhibitor for H_2_ production by *A. halophytica* was selected and then the effect of simazine concentration on H_2_ production by *A. halophytica* was investigated. The concentrations of simazine were varied at 0, 0.05, 0.5, 5, 25, and 50 µM.

### 2.3 Measurement of Cell and Chlorophyll *a* Concentration

The concentrations of cell and chlorophyll *a* were determined after simazine treatment for 0, 2, 24, 48, 72, and 96 h. An aliquot of cell suspension samples was collected, and the cell number was counted using a hemocytometer under a compound light microscope (Nikon Eclipse Ci-L, Japan). Cell concentration was calculated as a unit of cell number per volume of cell suspension. To analyze chlorophyll *a* concentration, 1 mL of cell culture was harvested by centrifugation at 8,000 × g at 4°C for 10 min. Chlorophyll *a* was extracted by adding 1 ml of 90% (v/v) methanol to a cell pellet, subsequently vortexing and incubating at 25°C under darkness for 1 h. Chlorophyll *a* concentration of pigment extract was determined by measuring an absorbance at 665 nm (MacKinney, 1941).

### 2.4 Measurement of H_2_ and O_2_ Production

The measurement of H_2_ and O_2_ production was determined by analyzing 500 µL of gas phase in the headspace of a vial containing 5 ml of cell suspension using a gas chromatograph (Hewlett-Packard HP5890A, Japan) with a molecular sieve 5°A 60/80 mesh packed column by a thermal conductivity detector (Taikhao et al., 2013). H_2_ and O_2_ production was calculated in terms of H_2_ and O_2_ produced per dry cell weight per time (µmol H_2_ g^−1^ dry cell weight h^−1^ and µmol O_2_ g^−1^ dry cell weight h^−1^).

### 2.5 Bidirectional Hydrogenase Activity Assay

Bidirectional hydrogenase activity of *A. halophytica* was determined in the presence of sodium dithionite-reduced methyl viologen (Taikhao et al., 2015). One mL of cell culture was added to 1 ml of 25 mM phosphate buffer (pH 7.0) containing 2.5 mM methyl viologen and 10 mM sodium dithionite. The reaction mixture was incubated at 25°C under dark anaerobic conditions for 15 min before H_2_ measurement by gas chromatograph as previously described (Taikhao et al., 2015). Bidirectional hydrogenase activity was expressed as µmol H_2_ g^−1^ dry weight min^−1^.

### 2.6 Measurement of Photosynthetic O_2_ Evolution Rate and Dark Respiration Rate

Photosynthetic O_2_ evolution and dark respiration rates were analyzed using a Clark-type oxygen electrode (Hansatech, United Kingdom). The measurement was carried out at 25°C. For photosynthetic O_2_ evolution measurement, 2 mL of *A. halophytica* cell suspension was added to a chamber and incubated in the dark for 15 min, prior to illumination under white-light intensity of 300 µmol photons m^−2^ s^−1^ for 15 min. The O_2_ evolution rate of cells was expressed as µmol O_2_ evolved per gram of cell dry weight per min. For dark respiration measurement, 2 mL of *A. halophytica* cell suspension in a chamber was incubated under a white-light intensity of 30 µmol photons m^−2^ s^−1^ for 15 min. Then, cells were incubated for 15 min under dark condition. The O_2_ consumption rate of cells was expressed as µmol O_2_ consumed per gram of cell dry weight per min.

### 2.7 Long-Term H_2_ Production Measurement

*A. halophytica* grown in BG11 (pH 7.4) supplemented with Turk Island salt solution for 7 days was harvested by centrifugation at 8,000 × g at 4°C for 10 min and resuspended in BG11_0_ supplemented with Turk Island salt solution. Cells were shaken on a rotary shaker at 120 rpm at 30°C under light for 24 h, subsequently harvested by centrifugation at 8,000 × g at 4°C for 10 min, and resuspended in 5 ml of BG11_0_. Cell suspension was transferred into a 12-ml glass vial and treated with simazine at a final concentration of 25 µM. Cells were purged with argon gas for 10 min and then incubated at 30°C under light and dark conditions. Long-term H_2_ production by *A. halophytica* cells treated with 25 µM simazine was determined for 10 days. *A. halophytica* cells without 25 µM simazine treatment and 25 µM simazine without cells were used as controls.

### 2.8 Statistical Data Analysis

The data in this study were statistically compared by a one-way analysis of variance (ANOVA) with Duncan’s multiple range test using IBM SPSS statistic 23 (IBM Corp, United States). Significant differences between treatments were considered at a level of 0.05 (*p* < 0.05).

## 3 Results

### 3.1 Screening of Inhibitors Affecting H_2_ Production by *A. halophytica*

In the first study, the measurement of H_2_ production by *A. halophytica* treated with various kinds of inhibitors at a final concentration of 5 µM was performed after dark and light incubation for 2 h. The results showed that under the light, *A. halophytica* treated with atrazine, DCMU, glyphosate, simazine, sodium azide, and 2,4-dinitrophenol showed significantly higher H_2_ production rate than cells without inhibitor treatment (Table 1). On the other hand, *A. halophytica* treated with atrazine, glyphosate, simazine, and rotenone under dark conditions showed a significantly higher H_2_ production rate than cells without inhibitor treatment (Table 1). *A. halophytica* cells treated without and with inhibitors under darkness notably produced 4–40 folds higher H_2_ than those under light (Table 1). Interestingly, the highest H_2_ production rates at 4.88 ± 0.45 and 46.22 ± 2.20 µmol H_2_ g^−1^ dry weight h^−1^ were obtained in cells treated with 5 µM simazine under light and dark conditions, respectively (Table 1). H_2_ production of cells treated with simazine under light and darkness was approximately 9 and 3 folds higher than those without simazine treatment, respectively.

**TABLE 1:** Effect of various types of inhibitors on H_2_ production rate of *Aphanothece halophytica* after 2 h of incubation under the light and darkness. The concentration of all inhibitors used in this study was 5 µM. Data are presented as means ± SD (*n* = 3). Different letters in columns indicate a significant difference, and the same letter indicates no significant difference according to Duncan’s multiple range test at *p* < 0.05.

Type of inhibitor	H_2_ production rate (μmolH_2_ g^−1^ dry weight h^−1^)	
	Light condition	Dark condition
Control	0.55 ± 0.07^d^	15.55 ± 0.95^def^
Atrazine	3.05 ± 0.35^b^	23.93 ± 0.17^c^
DCMU	1.623 ± 0.13^c^	16.98 ± 0.99^de^
Glyphosate	3.20 ± 0.27^b^	36.66 ± 1.62^b^
Simazine	4.88 ± 0.45^a^	46.22 ± 2.20^a^
Malonic acid	0.76 ± 0.13^d^	15.02 ± 1.78^ef^
Rotenone	0.67 ± 0.06^d^	25.95 ± 0.89^c^
Sodium azide	1.89 ± 0.37^c^	18.49 ± 2.54^d^
2,4-Dinitrophenol	2.83 ± 0.41^b^	12.48 ± 2.06^f^
DL-Glyceraldehyde	0.52 ± 0.21^d^	13.37 ± 1.94^ef^
Sodium arsenate	0.98 ± 0.11^d^	15.82 ± 0.93^def^

### 3.2 Effects of Simazine Concentration on Cell Concentration and Chlorophyll *a* Content

**FIGURE 1:**
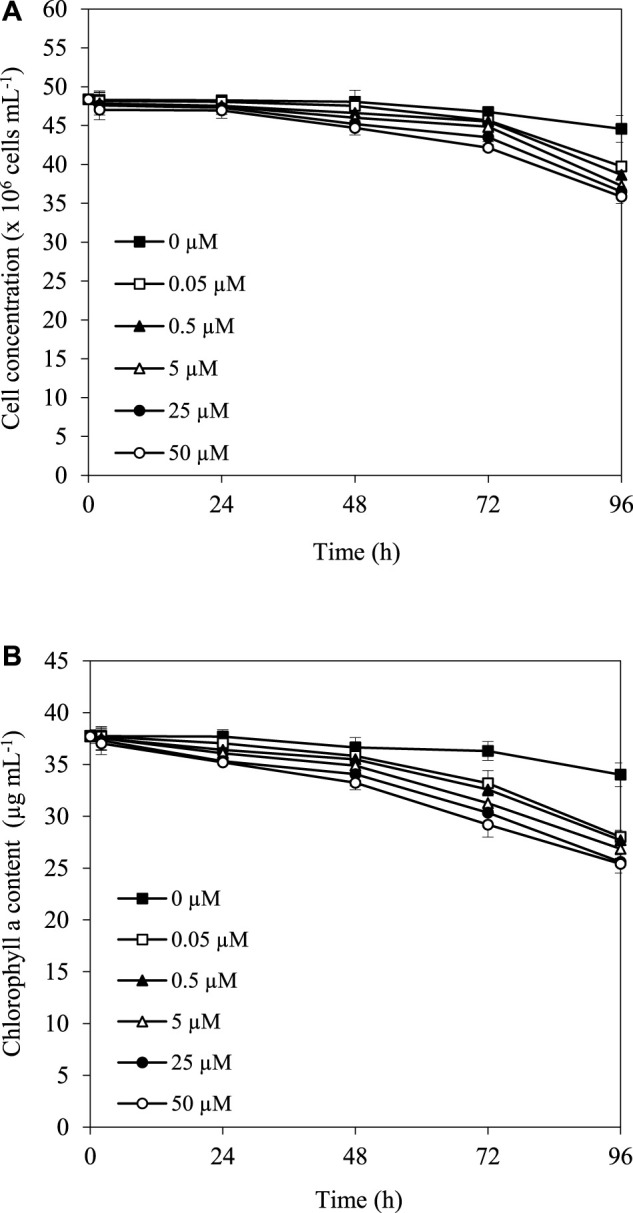
Effect of simazine concentration on cell concentration **(A)** and chlorophyll *a* content **(B)** of *Aphanothece halophytica* after various incubation durations.

The herbicide simazine functions as a photosynthetic inhibitor, which might affect the growth and pigment content, especially chlorophyll content, in cyanobacterial strains. The results showed that after treatment with 0.05, 0.5, 5, 25, and 50 µM simazine at 30°C under the light, cell and chlorophyll concentrations of *A*. *halophytica* were slightly reduced in the first 24 h, and more reduction was observed after 48 h of treatment (Figure 1A,B). In addition, it is noted that the higher the simazine concentration, the stronger its effect on the reduction of cell concentration and chlorophyll content.

### 3.3 Effect of Simazine Concentration on H_2_ and O_2_ Production

**FIGURE 2:**
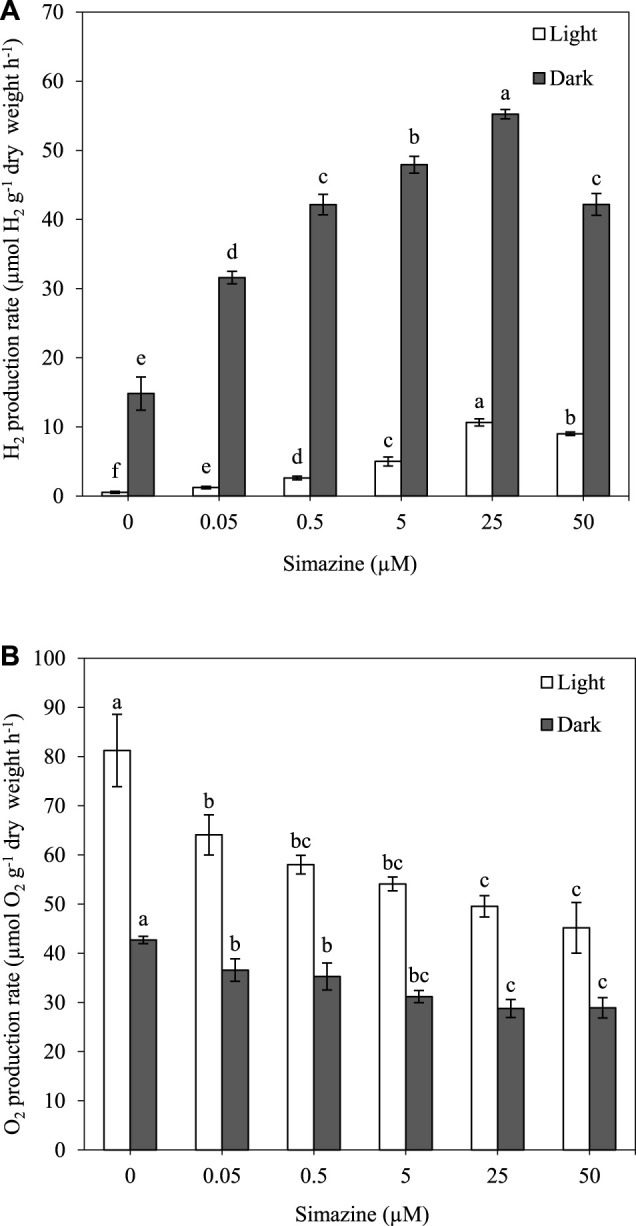
Effect of simazine concentration on H_2_ production rate **(A)** and O_2_ production rate **(B)** by *Aphanothece halophytica* after 2 h of incubation under the light (open square) and under darkness (solid square). Data are presented as means ± SD (*n* = 3). Different letters above the columns indicate a significant difference according to Duncan’s multiple range test at *p* < 0.05.

H_2_ and O_2_ production were measured in *A. halophytica* treated with various concentrations of simazine (0.05–50 µM) anaerobically under light and dark conditions at 30°C for 2 h. The results showed that a higher concentration of simazine increased H_2_ production but decreased O_2_ production under both light and dark conditions (Figure 2A,B). Cells treated with 25 µM simazine under light and dark conditions had an H_2_ production rate of 10.65 ± 0.53 and 55.23 ± 0.67 µmol H_2_ g^−1^ dry weight h^−1^, accounting for approximately 20 and 4 folds higher production than those without simazine treatment, respectively (Figure 2A). At 50 µM simazine, a significant decrease in H_2_ production rate was observed (Figure 2A). Moreover, cells treated without and with all concentrations of simazine under darkness produced higher H_2_ concentrations than those in light (Figure 2A).

### 3.4 Effect of Simazine Concentration on Bidirectional Hydrogenase Activity, Photosynthetic O_2_ Evolution and Dark Respiration

The measurement of bidirectional hydrogenase activity, photosynthetic O_2_ evolution, and dark respiration was performed in *A. halophytica* cells adapted to BG11_0_ for 24 h prior to a treatment with various concentrations of simazine under the light for 2 h. An increase in simazine concentration resulted in a significant increase in bidirectional hydrogenase activity and dark respiration rate but a decrease in photosynthetic O_2_ evolution (Table 2). The highest bidirectional hydrogenase activity at 53.64 ± 1.81 µmol H_2_ g^−1^ dry weight min^−1^ was observed in cells treated with 25 µM simazine (Table 2). Cells treated with 50 µM simazine showed lower bidirectional hydrogenase activity than those with 25 µM simazine (Table 2). This corresponded with the results of H_2_ production seen in Figure 2.

**TABLE 2:** Effect of simazine concentrations on bidirectional hydrogenase activity, photosynthetic O_2_ evolution, and dark respiration rate of *Aphanothece halophytica* after 2 h of treatment under the light. Data are presented as means ± SD (*n* = 3). Different letters on the columns indicate the significant difference, and the same letter indicates no significant difference according to Duncan’s multiple range test at *p* < 0.05.

Simazine (µM)	Bidirectional hydrogenase activity (μmol H_2_ g^−1^ dry wt min^−1^)	Photosynthetic O_2_ evolution (μmol O_2_ g^−1^ dry wt min^−1^)	Dark respiration rate (μmol O_2_ g^−1^ dry wt min^−1^)
0	13.36 ± 0.63^e^	824.00 ± 37.73^a^	186.77 ± 2.48^d^
0.05	22.46 ± 0.67^d^	636.04 ± 39.29^b^	210.77 ± 7.85^c^
0.5	37.55 ± 0.98^c^	562.95 ± 8.61^c^	221.76 ± 3.62^bc^
5	45.53 ± 0.48^b^	169.59 ± 10.62^d^	232.79 ± 3.93^b^
25	53.64 ± 1.81^a^	64.70 ± 8.07^e^	252.62 ± 7.85^a^
50	45.26 ± 0.98^b^	48.49 ± 6.40^e^	191.84 ± 6.52^e^

### 3.5 Long-Term Dark Fermentative H_2_ Production

**FIGURE 3:**
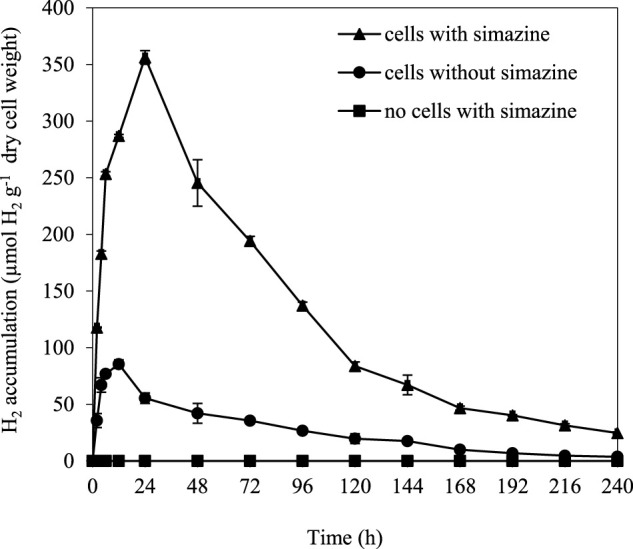
Long-term H_2_ accumulation of *Aphanothece halophytica* treated with and without 25 µM simazine during 10 days of dark anaerobic incubation. *Aphanothece halophytica* grown in BG11 for 7 days was harvested by centrifugation and suspended in BG11_0_. Cells were incubated in BG11_0_ under the light for 24 h before treatment with 25 µM simazine. Cells were purged with argon for 10 min and incubated at 30°C for 10 days under darkness. Cells without simazine treatment and simazine without cells were used as controls.

Long-term dark fermentative H_2_ production was determined in *A. halophytica* cells treated with and without 25 µM simazine for 10 days. The results showed that *A. halophytica* had maximum H_2_ accumulation with 356.21 ± 6.04 µmol H_2_ g^−1^ dry weight when treating cells with 25 µM simazine under dark anaerobic condition at 24 h (Figure 3). The maximum H_2_ accumulation was approximately 4 folds higher than that of cells without simazine treatment. No H_2_ production was observed in the negative control containing only 25 µM simazine.

## 4 Discussion

Simazine has been shown as a popular algicide for controlling the growth of both unicellular and attached filamentous algae (Snow, 1963). It can inhibit photosynthetic electron transport by binding to the plastoquinone B (Q_B_) binding site on the D1 protein of PS II, resulting in an interruption of CO_2_ fixation and production of ATP and NAD(P)H (Ahrens, 1994). Until now, only few studies have reported on the effect of simazine on H_2_ production by cyanobacteria. Previously, the N_2_-fixing filamentous cyanobacterium *Nostoc muscorum* treated with 2 µM simazine in the light produced approximately 2 folds higher H_2_ than the untreated cells. However, simazine did not affect acetylene reduction or nitrogenase activity in this cyanobacterium. The increased H_2_ production was due to the partial lowering of O_2_ in the cell, thus preventing oxidative H_2_ consumption (Spiller et al., 1978).

In this study, other inhibitors for photosynthetic electron transport such as atrazine and glyphosate could also induce H_2_ production by *A. halophytica* under both light and dark conditions (Table 1), indicating that they could inhibit electron transport in photosynthetic and/or other metabolic pathways. This reduces the number of electrons in photosystem II activity, resulting in a decrease in O_2_ evolution and finally promoting H_2_ production. Atrazine inhibits photosynthetic activity by blocking electron transport during the Hill reaction of PSII (Suresh Kumar et al., 2014). However, atrazine affects algae with a wide variety of responses depending on concentrations, duration of exposure, and type of algal species (Tang et al., 1998). Similarly, glyphosate was found to interrupt the photosynthetic electron transport and O_2_ evolution in both wild-type and mutant cells of *Anabaena doliolum* (Shikha and Singh, 2004). Although these inhibitors play a similar role in interrupting photosynthesis, they show such a difference in affinity for binding substrate and inhibitory activity.

Interestingly, DCMU induced H_2_ production by *A. halophytica* only upon illumination (Table 1). On the other hand, a known photosystem II inhibitor, carbonyl cyanide *m*-chlorophenyl hydrazine (CCCP), has been reported to induce H_2_ production by *A. halophytica* under both light and dark conditions (Pansook et al., 2019b). This is because photosystem II, a target of inhibition by DCMU, is functional upon illumination, whereas CCCP acts not only as a photosystem II inhibitor but also as an uncoupling agent of oxidative phosphorylation, which takes place under both illumination and darkness. The previous study showed that H_2_ production by *Oscillatoria chalybea* and *Synechocystis* sp. PCC6803 was enhanced in cells treated with 5 µM CCCP (Abdel-Basset and Bader, 1998), whereas the marine green alga *Platymonas helgolandica* var. *tsingtaoensis* increased H_2_ production due to the complete PSII inhibition by 50 µM DCMU (Zhang et al., 2012). Likewise, the cyanobacterium *Anabaena cylindrica* treated with 1.0 µM DCMU enhanced H_2_ production, which was partly due to the low level of O_2_ content (Chen et al., 2013), and DCMU at 10 mM increased 1.5-fold H_2_ production by the cyanobacterium *Desertifilum* sp. IPPAS B-1220 (Kossalbayev et al., 2020). Other inhibitors including the respiration inhibitor malonic acid, a CO_2_ fixation inhibitor glyceraldehyde, and a Krebs cycle inhibitor sodium arsenate did not induce H_2_ production rate by *A. halophytica*, suggesting no involvement of these inhibitors in H_2_ metabolism. Some inhibitors, such as a respiration inhibitor sodium azide and an inhibitor of uncoupling agent of oxidative phosphorylation 2,4-dinitrophenol (DNP), induced H_2_ production only under light condition, whereas a respiration inhibitor rotenone induced H_2_ production only under dark condition (Table 1). It was previously reported that *Cyanothece* sp. could increase the H_2_ production rate in cells treated with 50 µM 2,4-dinitrophenol under darkness (Skizim et al., 2012).

Normally, simazine concentrations used for controlling algal and cyanobacterial growth range from 0.5 to 1.0 mg L^−1^ or from 2.5 to 5 µM (Suresh Kumar et al., 2014). In this study, a high concentration of simazine and long-term incubation resulted in a decrease in cell and chlorophyll concentrations (Figure 1A,1B). These results indicated the toxicity of simazine due to the inhibition of electron transport in photosynthesis, thus resulting in a decrease in cell concentration and intracellular pigment contents, especially chlorophyll *a*. These results are in line with the previous study on filamentous cyanobacteria *Anabaena circinalis* and *Anabaena variabilis*; and green algae *Protosiphon botryoides* and *Spirogyra jurgensii*, showing that simazine inhibited their growth and reduced chlorophyll content (O’Neal and Lembi, 1983; Millie et al., 1992; Kobbia et al., 2001). In this study, simazine exhibited toxicity to cell growth and affected intracellular pigments of *A. halophytica* under long-term treatment.

Our results indicated the importance of simazine concentration on photosynthetic activity and H_2_ metabolism of *A. halophytica* cells. A high concentration of simazine caused a decrease in O_2_ concentration due to O_2_ evolution *via* oxygenic photosynthesis and an activation of dark respiration rate. The decrease in O_2_ concentration promoted bidirectional hydrogenase activity, leading to an increase in H_2_ production. Under the light, simazine was shown to interrupt photosynthetic electron transport at photosystem II by displacing Q_B_ from its binding site on the D1 protein (O’Neal and Lembi, 1983), resulting in an inhibition of photosynthetic activity or photosynthetic O_2_ evolution. This caused a reduction in O_2_ concentration, thereafter promoting bidirectional hydrogenase activity. Under dark anaerobic conditions, where photosynthetic activity is inactive, simazine promoted the dark respiration rate, providing higher O_2_ consumption and higher level of NAD(P)H from carbohydrate degradation. The lower O_2_ concentration activated bidirectional hydrogenase activity, and NAD(P)H gave electrons to hydrogenase, finally resulting in higher H_2_ production by *A. halophytica*. Since *A. halophytica* produces H_2_ under dark anaerobic conditions *via* a catabolism of storage glycogen (Taikhao et al., 2015), the effect of simazine on glycogen content was investigated. Unfortunately, no significant differences in the glycogen content of cells treated and untreated with 25 µM simazine were found (data not shown). Apart from being a photosystem II inhibitor, simazine might affect other metabolisms, including H_2_ metabolism. In *Protosiphon botryoides* and *Anabaena variabilis*, simazine treatment was shown to increase respiration rate (Kobbia et al., 2001). In oat plant (*Avena sativia* L. var. Seminole), simazine treatment showed a decrease in protein synthesis under darkness (Singh and West, 1967). However, too high a concentration of simazine (50 µM) is likely to be toxic to *A. halophytica* cells. This study confirmed the capability of the effective inhibitor simazine for enhancement of dark fermentative H_2_ production by *A. halophytica*. However, during long-term incubation after 24 h of simazine treatment, cells reduced dark fermentative H_2_ production due to the decreased action of simazine and the toxicity of simazine to cyanobacterial growth and cellular metabolism (O’Neal and Lembi, 1983).

## 5 Conclusion

The photobiological H_2_ production by *A. halophytica* was significantly enhanced by treatment with atrazine, DCMU, glyphosate, simazine, sodium azide, and 2,4-dinitrophenol, whereas the dark fermentative H_2_ production was significantly increased in cells treated with atrazine, glyphosate, simazine, and rotenone. Among all the inhibitors, simazine is the best inhibitor to enhance H_2_ production by *A. halophytica* under both light and dark conditions. High simazine concentration and long-term incubation reduced cell concentration and chlorophyll content due to its cell toxicity. The optimal concentration of simazine for H_2_ production by *A. halophytica* was 25 µM. Simazine treatment reduced photosynthetic O_2_ evolution, resulting in an increase in bidirectional hydrogenase activity. In addition, simazine induced O_2_ consumption by enhancing the dark respiration rate. These incidences promoted H_2_ production in *A. halophytica*.

## Data Availability

The original contributions presented in the study are included in the article/Supplementary Material; further inquiries can be directed to the corresponding author.
